# *Blumea htamanthii* (Asteraceae), a new species from Myanmar

**DOI:** 10.3897/phytokeys.138.38815

**Published:** 2020-01-10

**Authors:** Yulan Peng, Chenxuan Yang, Yan Luo

**Affiliations:** 1 Key Laboratory of Mountain Ecological Restoration and Bioresource Utilization & Ecological Restoration Biodiversity Conservation, China Key Laboratory of Mountain Ecological Restoration and Bioresource Utilization & Ecological Restoration Biodiversity Conservation Chengdu China; 2 Key Laboratory of Sichuan Province, Chengdu Institute of Biology, Chinese Academy of Sciences, P.O. Box 416, Chengdu, Sichuan 610041, China Chengdu Institute of Biology, Chinese Academy of Sciences Chengdu China; 3 Gardening and Horticulture Department, Xishuangbanna Tropical Botanical Garden, Chinese Academy of Sciences, Menglun, Mengla, Yunnan 666303,China Xishuangbanna Tropical Botanical Garden, Chinese Academy of Sciences Menglun China

**Keywords:** Asteraceae, *Blumea htamanthii*, Myanmar, new species

## Abstract

A new species, *Blumea htamanthii* Y.L. Peng, C.X. Yang & Y. Luo from Myanmar is described. The new species is distinguished from *B. bifoliata* by its leaves with short petioles, abaxially purple, leaf blade with papillary hair and sparse multicellular villous, capitula with 1–4 heads, glabrous florets and usually unbranched stems. A key to *Blumea* species in Myanmar is provided.

## Introduction

*Blumea* DC. is one of the largest and most taxonomically difficult genera in the Tribe Inulaceae, which includes approximately 100 species worldwide (Randeria 1960; Anderberg 1991, 1994, 2009; Pornpongrungrueng et al. 2016). Blumea is a monophyletic genus supported by molecular data (Pornpongrungrueng et al. 2007, 2009). *Blumea* is primarily distributed in tropical Asia, Africa and Oceania, while its highest diversity is in tropical Asia (Pornpongrungrueng et al. 2016). With the exception of the most common weeds in disturbed habitat, some species of *Blumea* have very narrow distribution areas at the edge of the forest. With the exception of partial revisions of 49 species of *Blumea* throughout the whole world by Randeria (1960) and of 27 species in continental Southeast Asia by Pornpongrungrueng et al. (2016), a whole revision of this genus worldwide is still lacking. Seventeen species were reported from Myanmar (Kress et al. 2003).

## Material and methods

During our fieldwork in Myanmar in 2019, we found an undescribed species of *Blumea.* The plant that we collected in the Htamanthi Wildlife Reserve, Sagaing, is easily distinguished from any other taxa of *Blumea* by its specific flowers and leaves, i.e. its basal rosette abaxially purple leaves with papillary hairs and multicellular villous and 1–4 capitula at the ends of the peduncles and unribbed achenes.

## Results

### Blumea htamanthii

Taxon classificationPlantaeAsteralesAsteraceae

Y.L.Peng, C.X.Yang & Y.Luo sp. nov.

6EF01D16-1467-56EE-920D-572BF5A71C5F

urn:lsid:ipni.org:names:77204219-1

Figs 1 , 2

#### Diagnosis.

This new species is the most similar to *Blumea bifolia* (Linn.) DC. in its obovate-oblong leaves, reflexed linear phyllaries, flat, alveolate, glabrous receptacles. However, it is distinguished by its leaf blades with papillary hairs and sparse multicellular villous, abaxial purple, 1–4 capitula at the ends of the peduncles and its unribbed achenes.

#### Type.

**Myanmar**: Htamanthi Wildlife Sanctuary, Hkamti District of Sagaing Region, the cliff near the edges of the forest along the branch river of Chindwin River, elevation 127 m, 25.4948593°N, 95.4319749°E , May 23 2019,Y.L. Peng, C.X. Yang & Y. Luo, SE02614 (Holotype CDBI!, Isotype HITBC!, RAF!).

#### Description.

Annual herbs, herbaceous, 5–25 cm tall. Stems erect, occasionally procumbent, villous with multicellular hairs, leaves basal rosette or sub-basal rosette and a few cauline, petioles 0.2 to 0.3 cm long, at the base of petioles with white pilose hairs, lamina obovate or obovate-oblong, thinly papyraceous, 0.9–3.5 × 0.3–1.2 cm, acute at the apex, base abruptly constricted into winged petiole, margins distantly dentate, villous with multicellular hairs, both surfaces hairy, significantly discoloured, upper surface bright green, leaf blade with papillary hairs and sparse multicellular pilose, multicellular pilose on the veins are dense, lower surface purple, the base of margins serrate, apex acute; inflorescences loose panicles, 3–10 cm long, capitula terminal, rarely axillary, 1–4 heads at the ends of the peduncles, 4–6 mm in diameter, peduncles 5–25 mm long with white pilose hairs; phyllaries herbaceous, slightly longer than the florets, 10–20 mm long, phyllaries in 5 (–6)-seriate, reflexed, outer phyllaries linear, with colleters and pilose hairs, lower part of the inner phyllaries lanceolate, upper part abruptly reduced to a linear tip, the middle and upper part margin of the inner phyllaries lacerate, with sparse multicellular hairs, receptacle 0.5–1 mm in diameter, flat or slightly convex, alveolate, glabrous. Florets yellow, tubular, glabrous; those of the bisexual florets, corolla tube 3.5–4.5 mm long, with 5 ovate, papillate lobes, styles of the hermaphroditic flowers are wrapped in a slightly longer stamen tube; those of the female florets are filiform, up to 3.5 mm long, with 2 to 3 lobed, corolla tube 1–1.5 mm long. Cypselas pale brown, oblong, pubescent, not ribbed, 0.4–0.6 mm long, pilose; pappus carducous, white, 3–4 mm long.

**Figure 1.**
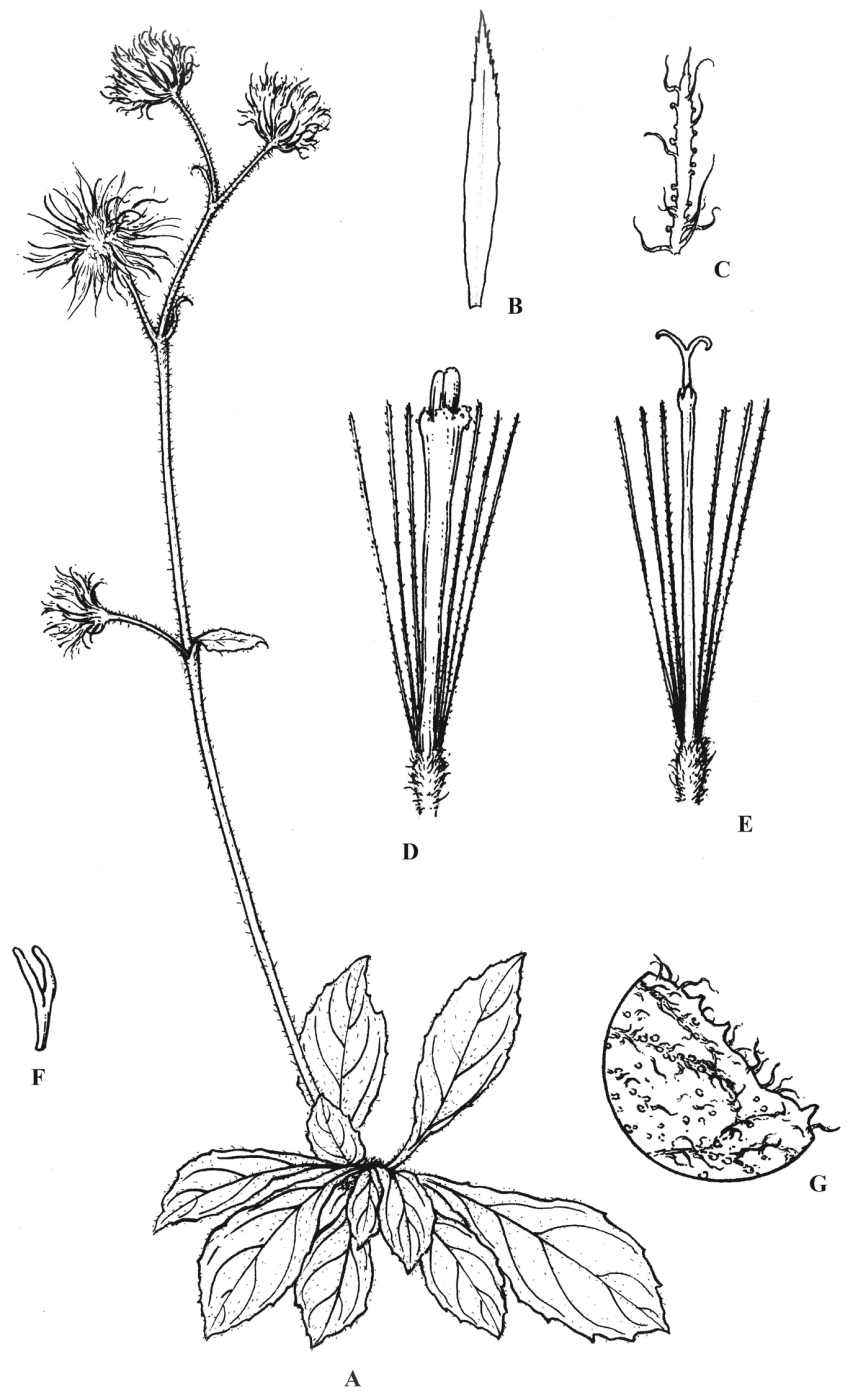
*Blumea htamanthii* Y.L.Peng, C.X.Yang & Y.Luo, sp. nov. **A** habit **B** the inner phyllary **C** the outer phyllary **D** bisexual floret **E** female floret **F** style of bisexual floret **G** magnified part of upper surface of the leaf. Drawings: Jian Gu based on the holotype.

**Figure 2.**
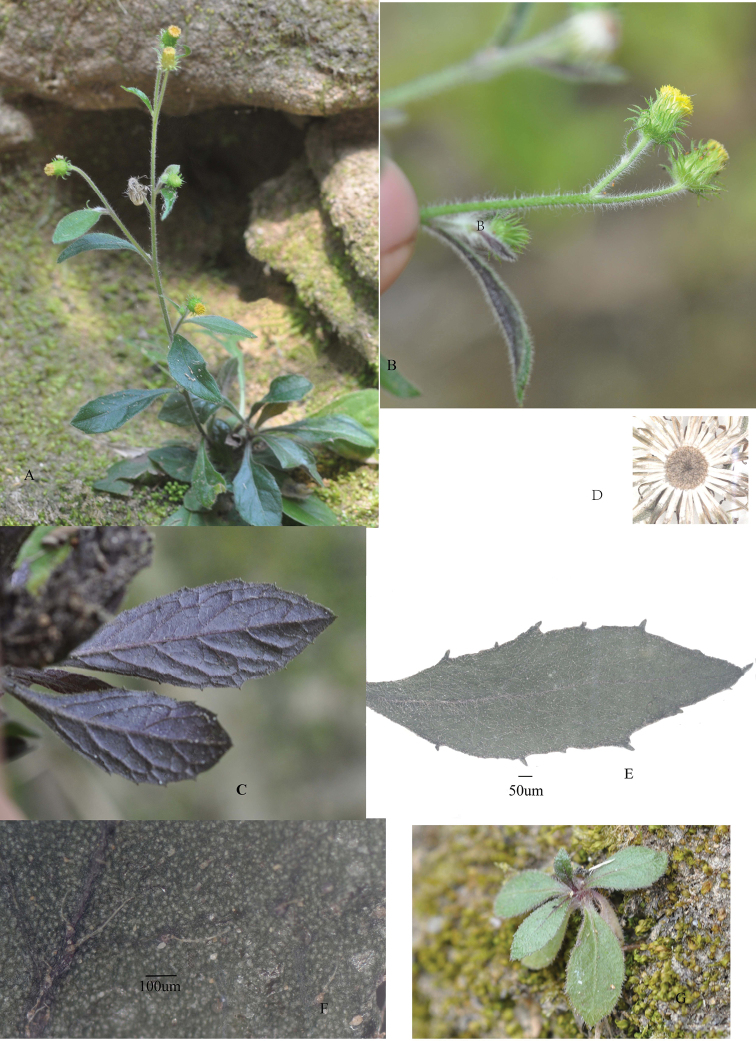
*Blumea htamanthii* Y.L.Peng, C.X.Yang & Y.Luo , sp. nov. in the field and magnified leaves and receptacle **A** the whole plant **B** inflorescences **C** the abaxial surface of the leaf **D** receptacle **E** the upper surface of the leaf **F** magnified part of upper surface of the leaf **G** basal leaves. Photos: Y.L. Peng.

#### Etymology.

The new specific epithet “htamanthii” refers to the name of the town along the Chindwin River, Hkamti District of Sagaing, Myanmar, where the novel species was discovered.

#### Phenology.

Flowering and fruiting April to June.

#### Distribution and habitat.

Myanmar. Sagaing, Htamanthi; *Blumea htamanthii* is only known from the type collection along the branch river of Chindwin River, growing on the steep rocks near the forest from 66–366 m altitude above mean sea level, 25.4948°–25.5152°N, 95.4319°–95.5268°E in the Htamanthii Nature Reserve.

#### Additional material examined.

25.4947931°N, 95.4319147°E, elevation 121–129 m, 23 May 2019,Y.L Peng, C.X. Yang &Y. Luo SE02645, SE02694 (CDBI, HITBC, RAF); 25.5132139°N, 95.5269449°E, elevation 36–367 m, 26 May 2019, Y.L. Peng, C.X. Yang & Y. Luo SE02730, SE02731, SE02736 (CDBI, HITBC, RAF); 25.5127053°N, 95.5267582°E, elevation 366 m, 27 May 2019, Y.L Peng, C.X. Yang & Y. Luo, SE02769 (CDBI, HITBC, RAF); 25.5128305°N, 95.5268144°E, elevation 366 m, 27 May 2019, SE02770 (CDBI, HITBC, RAF), 25.5133152°N, 95.5262927°E, elevation 340 m, 27 May 2019, Y.L Peng, C.X. Yang & Y. Luo SE02777 (CDBI, HITBC, RAF); 25.5128089°N, 95.5266037°E, elevation 160 m, 27 May 2019, Y.L Peng, C.X. Yang & Y. Luo SE02806, SE02861(CDBI, HITBC, RAF).

#### Discussion.

*Blumea htamanthii* resembles *B. bifoliata* (Linn.) DC. and *B. diffusa* R. Br. ex Benth. in its reflexed linear phyllaries, flat, alveolate, glabrous receptacle and obovate leaves. *Blumea htamanthii* differs from *B. bifoliata* by erect stem and basal bicolour rosette leaves, abaxially purple, with short petioles, leaf blade with papillary hairs and sparse multicellular villous and 1–4 capitula at the ends of the peduncles, achenes not ribbed (vs. leaves sessile, one colour, villous with multicellular hairs and stipitate glands, solitary capitula, achenes 6–10 ribbed) (Table 1). *Blumea htamanthii* differs from *B. diffusa* in erect stems and leaves with short petioles, cauline leaf base not amplexicaulous, and 1–4 capitula at the ends of the peduncles (vs. stems procumbent, leaves sessile, one colour, cauline amplexicaulous, solitary capitula) (Table 1). In addition, *B. bicolor* is endemic in the Philippines with abaxially purple leaves (Merrill 1912, Randeria 1960). However, it is a tall erect herb with leaves aggregated in the middle portion of the stem, leave blade oblong elliptic, 5.5–23.5 × 1.3–8.6 cm and achenes ribbed. Its morphological traits are significantly different from those of *B. htamanthii* (Table 1).

**Table 1.** A list of the morphological differences between *Blumea tamanthii*, *B. bifoliata*, *B. diffusa* and *B. bicolor*.

Characters	*Blumea htamanthii*	*Blumea bifoliata*	*Blumea diffusa*	*Blumea bicolor*
Leaf arrangement patterns	Basal rosette or sub-basal rosette and a few cauline, the cauline ones are all alternate	Mostly cauline, the uppermost pair are subopposite	Basal rosette or sub-basal rosette and a few cauline; the cauline ones are all alternate and amplexicaulous	The lower part of the stem naked, leafless; the leaves mostly aggregated in the middle portion of the stem
Leaf morphology	Petioles 0.2 to 0.3 cm long, lower surface purplish, apex acute, leaf blade obovate or obovate-oblong, villous with papillary hair and sparsely multicellular villous, 0.9–3.5× 0.3–1.2 cm	Sessile, both surfaces green, the apex acute or apiculate, leaf blade oblong or ovate, villous with multicellular hairs and stipitate glands, radical leaves 0.7–3 × 0.4–1.5 cm	Sessile, both surfaces green, the apex acute to apiculate, obovate or rarely oblanceolate, pilose with colleters and multicellular hairs, 2–6 cm × 1.0–2.5 cm wide	Lower surface purplish, apex sharply acuminate, blade oblong elliptic, sparsely pilose with multicellular hairs, 5.5–23.5× 1.3–8.6 cm
Stem	Erect, occasionally procumbent, usually unbranched, pilose with long, white hairs	Erect, branched from the base, ascending or rarely procumbent	Procumbent, stems branched from the base, pilose with long, white hairs	Erect, generally unbranched, puberulous
Capitula	1–4, colleters and pilose on the outer phyllaries, the middle and upper part margin of the inner phyllaries lacerate	1, glands on the phyllaries	1, pilose on the phyllaries	Several formed a lax, terminal panicle, pubescent on the phyllaries
Florets	Glabrous	Sparsely pubescent on the lobes	Glabrous	Bisexual florets pubescent, female florets glabrous
Cypselas	Pilose, not ribbed	Pilose, 6–10-ribbed	Sparsely pilose, 10-ribbed	Ribbed, pubescent

##### Key to *Blumea* species in Myanmar (including the closely related species *B. bicolor* in the Philippines and *B. diffusa* in Australia)

**Table:** 

1a	Plants densely white-woolly	**2**
2a	Outer phyllaries oblong-lanceolate, acute	***Blumea hieraciifolia* (Don) DC.**
2b	Outer phyllaries linear and tapering	**3**
3a	Capitula in large lax panicles; pappus red; corolla lobes of bisexual florets glabrous	***Blumea densiflora* DC.**
3b	Capitula in compact, spiciform panicles; pappus white; corolla lobes of bisexual florets hairy	***Blumea lacera* (Burm. f.) DC.**
1b	Plants glabrous or variously pubescent	**4**
4a	Phyllaries at least the outer phyllaries, oblong-ovate to oblong-lanceolate	**5**
5a	Climber; receptacle densely pubescent; corolla lobes of female florets with multicellular hairs	***Blumea riparia* (Blume) DC.**
5b	Erect, receptacle fimbrillate or rarely pilose; corolla lobes of female florets glabrous	**6**
6a	Receptacle fimbrillate or rarely pilose glabrous	***Blumea lanceolaria* (Roxb.) Druce**
6b	Receptacle pilose	**7**
7a	Capitula in narrow panicles	***Blumea hirsuta* (Less.) M. R. Almeid**
7b	Capitula in large, spreading panicles	***Blumea repanda* (Roxb.) Hand.-Mazz**
4b	Phyllaries all linear or linear-lanceolate	**8**
8a	Receptacle fimibrillate	***Blumea aromatica* DC.**
8b	Receptacle glabrous or pilose.	**9**
9a	Leaves purplish on abaxial surface	**10**
10a	Height 20–100 cm, the lower part of the stem naked, leafless, mostly aggregated in the middle portion of the stem	***Blumea bicolor* Merr**
10b	Height 5–25 cm, leaves basal rosette or sub-basal rosette and a few cauline	***Blumea htamanthii* Y.L.Peng, C.X.Yang & Y.Luo, sp. nov.**
9b	Leaves not purplish on abaxial surface	**11**
11a	Pappus reddish	***Blumea balsamifera* (L.) DC.**
11b	Pappus white	**12**
12a	Capitula solitary, axillary and terminal	**13**
13a	Diffuse herbs; leaves mostly radical, the cauline ones all alternate	***Blumea diffusa* R. Br. ex Benth**
13b	Erect herbs; leaves mostly cauline, the uppermost pair subopposite	***Blumea bifoliata* (L.) DC.**
12b	Capitula interruptedly spiciform paniculate or loose or dense paniculate	**14**
14a	Inflorescence an interrupted spiciform panicle	***Blumea fistulosa* (Roxb.) Kurz**
14b	Inflorescence a loose or dense paniculate	**15**
15a	Leaves spinous-toothed, stems procumbent	***Blumea oxyodonta* DC.**
15b	Leaves not spinous-toothed, stems erect	**16**
16a	Receptacle minutely pilose	**17**
17a	Leaves not lyrately lobed	***Blumea adenophora* Franch**
17b	Leaves lyrately lobed	***Blumea sinulata* (Lour.) Merr**
16b	Receptacle glabrous	**18**
18a	Achenes ribbed	**19**
19a	Plants more or less glabrate	***Blumea virens* Wall. ex DC.**
19b	Plants pubescent or variously glandular	***Blumea napifolia* DC.**
18b	Achenes subangulate to terete	**20**
20a	Leaves usually not lobed; corollas purple	***Blumea axillaris* (Lam.) DC.**
20b	Leaves often lyrately lobed; corollas yellow	***Blumea lacera* (Burm. f.) DC.**

## Supplementary Material

XML Treatment for Blumea htamanthii
